# Effect of Mechanical Grinding on the Physicochemical, Structural, and Functional Properties of Foxtail Millet (*Setaria italica* (L.) P. Beauv) Bran Powder

**DOI:** 10.3390/foods11172688

**Published:** 2022-09-03

**Authors:** Kehong Liang, Hong Zhu, Yue Zhang

**Affiliations:** 1 Institute of Food and Nutrition Development, Ministry of Agriculture and Rural Affairs, Beijing 100081, China; 2 College of Engineering, China Agricultural University, Bejing 100083, China

**Keywords:** foxtail millet bran, particle size, physicochemical property, structural property, antioxidant capacity

## Abstract

This study investigated the functional, physicochemical, and structural characteristics of foxtail millet bran powder with different particle sizes. The morphological analysis revealed that the surface roughness declined in conjunction with the particle sizes of the millet bran powder. The Fourier-transform infrared (FTIR) spectra showed that none of the samples generated any additional chemical functional groups. A decrease in the particle sizes of the millet bran powder increased their dissemination and surface areas, as well as the bulk density, tap density, water-holding capacity (WHC), angle of repose (θ) and angle of slide (α), and peak temperature, while the oil holding capacity (OHC) and crystallinity index (CI) value declined. Moreover, fine millet bran powder (54.7 μm) exhibited a higher protein, fat, soluble dietary fiber (SDF), total phenolic content, and antioxidant capacity than its coarse counterpart.

## 1. Introduction

The ancient Chinese grain, foxtail millet (*Setaria italica* (L.) P. Beauv.), is broadly distributed in the semi-arid and arid regions of the world [1]. Foxtail millet bran is a by-product of conventional milling that includes nutritional factors like dietary fiber, vitamins, fat, and protein [2]. Millet bran is higher in vitamins E, C, and B than the kernels, and represents 18% of the millet fiber content [3]. However, except for a minimal quantity that is converted into animal feed, the bran is mostly discarded, due to its undesirable taste. Furthermore, cereal brans, especially millet and rice brans, are often difficult to digest due to the low degradability of their fiber [4]. Studies have shown that even after mastication, the sizes of the wheat bran particles are barely altered and are not significantly digested before reaching the large intestine. The initial particle size of wheat bran is essential, since it enters the distal colon intact for fermentation [5].

The particle size can affect the physicochemical properties of powder [6,7], while the small particles, after processing, increase the particle surface area and cell wall degradation, promote phytochemical release [8], and improve particle biological activity. Zhang et al. [9] showed that particle size reduction is associated with increased angle of slide (α), angle of repose (θ), tap density, and bulk density. Moreover, bran particle size affects the bioaccessibility of phenolic acids [10]. Brewer et al. [11] observed an increase in phenolic compound extraction (such as phenolic acid, flavonoids, and carotenoids) and oxygen radical absorbance capacity (ORAC) after particle size reduction. Antioxidants are primarily found in the wheat bran aleurone fraction, increasing the extractable phenolic acids and phenolic content, as well as the antioxidant activity [12].

Minimal studies are available involving mechanical fragmentation to different degrees regarding the functional, structural, and the physicochemical characteristics of foxtail millet bran by-products. These properties influence the application value, ingredient extraction rate, and industrial production specifications of discarded millet bran. Consequently, this study investigates the impact of different particle sizes on the physicochemical, structural, and antioxidative properties of millet bran. The results are thoroughly analyzed and compared according to the different grinding scales. The findings may provide useful information about the application of foxtail millet bran in functional foods.

## 2. Materials and Methods

### 2.1. Materials

Commercial foxtail millet (*Setaria italica*) bran was supplied by the Jinan Longshan Millet Development Co., Ltd. (Jinan, Shandong, China).

### 2.2. Foxtail Millet Bran Powder Preparation

A high-speed crusher (As One Co., Ltd., Shanghai, China) was used to grind the foxtail millet bran samples, followed by mixing with stainless steel balls and smashing for 2 h. The powder was sieved using an electric, vibrating screening machine (8411, Shangyu Daoxu Xingfeng instrument factory, Shaoxing, Zhejiang, China), with standard mesh screen sizes of 60 (250 μm pore size), 80 (180 μm pore size), and 120 (125 μm pore size). The samples were sieved at above 60 mesh (M60), between 60 and 80 mesh (M60–80), between 80 and 120 mesh (M80–120), and below 120 mesh (M120).

### 2.3. The Main Chemical Components

The protein, carbohydrate, and fat content in the millet bran powder were determined according to the GB 5009 (2016) standard. The total (TDF), insoluble (IDF), and soluble dietary fiber (SDF) content was obtained according to the GB 5009.88-2014 standard. The total phenolic content was acquired according to a technique delineated by Chu et al. [13].

### 2.4. Antioxidant Activity Determination

The DPPH and ABTS scavenging activity assays were determined according to the modified methods of Wang et al. [14]. Here, 2.5 mL of a 38 μL DPPH methanolic solution and 0.5 mL of the respective extracts (the extraction was obtained by homogenizing 10 g millet bran and 100 mL ethanol) were mixed to determine the DPPH scavenging activity. The mixture was shaken and subjected to incubation for 30 min at room temperature in the dark, followed by an absorbance measurement at 517 nm. The scavenging activity (%) was calculated using Equation (1):

(1) $$\mathrm{DPPH}\;\mathrm{scavenging}\;\mathrm{activity}\;\left(\%\right)=\left(1-\frac{A_{1}-A_{2}}{A_{0}}\right)\times 100$$

For the ABTS scavenging activity assay, 50 μL of each extract was mixed separately with 3 mL of the ABTS • reagent (An equal volume of 7 mM (NH_4_)_2_ABTS aqueous solution was mixed with 2.45 mmol/L K_2_S_2_O_8_.). Prior to usage, the mixture was kept at room temperature in the dark for approximately 12 h. To obtain an absorbance of 0.70 ± 0.02 at 734 nm, the solution was diluted with ethanol. After 6 min of room-temperature incubation in the dark, the absorbance of the mixture was tested at 734 nm. The ABTS radical scavenging activity (%) was calculated using Equation (2):

(2) $$\mathrm{Scavenging}\;\mathrm{activity}\;\left(\%\right)=\left(1-\frac{A_{1}}{A_{0}}\right)\times 100$$

### 2.5. Bulk and Tap Densities

The volume of the powder (*V*_1_, mL) was measured after adding about 5 g (M_1_) to a volumetric cylinder (25 mL). Equation (3) was used to determine the bulk density (ρ_bulk_, ×10^3^ kg/m^3^).

(3) $$\rho_{bulk}=\frac{M_{1}}{V_{1}}$$

The tap density was calculated using the National Standard of the People’s Republic of China (21354-2008). The millet bran powder was weighed (M_2_, g) and tapped on a thick sponge until the decline in volume ceased. Then, Equation (4) was used to calculate the final powder volume (V_2_, mL) and obtain the ρ_tap_ (×10^3^ kg/m^3^).

(4) $$\rho_{tap}=\frac{M_{2}}{V_{2}}$$

### 2.6. Angle of Repose and Slide

The angle of repose (θ) and angle of slide (α) were measured using a previously described method [15]. For θ, a glass funnel was fixed vertically, 3 cm above the plane on the testbed. The powder was poured into the funnel until the formed cone made contact with the funnel end. The radius (R) and height (H) were recorded. The θ was calculated using Equation (5):

(5) $$\theta =\arctan\frac{H}{R}$$

The millet bran powder (about 5.0 g) was poured onto a glass plane and gradually tilted to facilitate particle sliding. The vertical distance (h) and plane length (L) were documented after stabilizing the powder cone. The α was obtained via Equation (6).

(6) $$\alpha =\arcsin\frac{h}{L}$$

### 2.7. Particle Size Distribution

The particle size dissemination of the millet bran powder was determined with a Mastersizer 3000 laser particle size instrument provided by Malvern Instrument Ltd. (Worcestershire, UK). The D_90_, D_50_, and D_10_, values represented cumulative percentiles of 90%, 50%, and 10% of the particle sizes below the values, respectively. The particle size width span was estimated using Equation (7). Equation (8) was used to determine the cell wall-breakage ratio (Φ).

(7) $$Span=\frac{D_{90}-D_{10}}{D_{50}}$$

(8) $$\phi =1-{\left(1-\frac{10}{D_{50}}\right)}^{3}$$

### 2.8. Color Analysis

A spectrocolorimeter (Labscan XE, Hunterlab, Reaton, VA, USA) was used to determine the powder color, and the results were presented according to the CIELAB color parameters. The criteria for the color analysis included b* (−60 to +60 blue to yellow), a* (−60 to +60 green to red), and L* (0–100 black to white). Furthermore, the ΔE was calculated with the powder as the control sample and determined using Equation (9):

(9) $$\Delta E=\sqrt{{\left(L^{*}-L_{0}^{*}\right)}^{2}+{\left(a^{*}-a_{0}^{*}\right)}^{2}+{\left(b^{*}-b_{0}^{*}\right)}^{2}}$$

### 2.9. Scanning Electron Microscopy (SEM)

The millet bran powder morphology was observed via SEM (SU 3500, Hitachi Co., Tokyo, Japan). The powder samples were gold-sprayed after they were secured using double-sided tape. A 10-kV acceleration voltage was used for SEM image capturing.

### 2.10. Hydration Property Analysis

#### 2.10.1. Water Holding Capacity (WHC)

Approximately 0.5 g of millet bran powder (W_1_) was mixed with 30 g of deionized water, soaked at 4 °C for 24 h, and centrifuged at 13,900× *g* for 15 min. Then, the residues were weighed (W_2_) after the removal of the supernatant. Equation (10) was used to determine the WHC:

(10) $$\mathrm{WHC}\left(g/g\right)=\frac{W_{2}-W_{1}}{W_{1}}$$

#### 2.10.2. Oil Holding Capacity (OHC)

Approximately 0.5 g of the sample (O_2_) and 10 mL of sunflower oil were mixed in a centrifuge tube and stored for 1 h at 25 °C. The supernatant was discarded following centrifugation for 15 min at 13,900× *g*, after which the residue weight (O_1_) was determined again. The OHC was calculated using Equation (11):

(11) $$\mathrm{OHC}\left(g/g\right)=\frac{O_{2}-O_{1}}{O_{1}}$$

### 2.11. Thermal Property Determination

Differential scanning calorimetry (DSC 214, NETZSCH Instruments, Bavaria, Germany) was used for thermal characterization. Here, 4–8 mg of the powder was placed in an aluminum pan, after which a microsyringe was used to add deionized water, yielding a water:flour ratio of 3:1. The temperature was increased from 20 °C to 120 °C at a nitrogen velocity of 50 cm^3^/min and a rate of 10 °C/min. The Universal Analysis 4.5A software (TA Instruments Ltd., New Castle, DE, USA) was employed to create the DSC curves.

### 2.12. Fourier Transform Infrared (FTIR) Analysis

The millet bran powder and potassium bromide (KBr) were mixed well and tested after being pressed into thin samples. An FTIR spectrometer (Nicolet iS5, Thermo Scientific, Waltham, MA, USA) was used to obtain the spectrum of each sample in a range of 4000 cm^−1^ to 400 cm^−1^ after 32 scans at a 4 cm^−1^ resolution.

### 2.13. X-ray Diffraction (XRD)

An X-ray diffractometer (XD-2, Beijing Purkinje General Instrument Co., Ltd., Beijing, China) was used to determine the XRD patterns at a working voltage of 36 kV and a 20mA current. The diffraction angle (2θ) was determined at a 1 °C/min scanning speed in a 10° to 40° range. The crystallinity index (CI) of the millet bran powder was determined via a technique delineated by Toba et al. [16]

### 2.14. Statistical Analysis

The experiments were all repeated three times, while the SPSS statistical software (Version 19.0, SPSS Inc., Chicago, IL, USA) was used for the statistical analysis. The differences between the samples were determined via one-way ANOVA, which was employed to demonstrate the differences between the samples, presenting the data as a mean ± standard deviation. A value of *p* < 0.05 was deemed statistically significant.

## 3. Results and Discussion

### 3.1. Characterization of the Structural Properties

**Figure 1:**
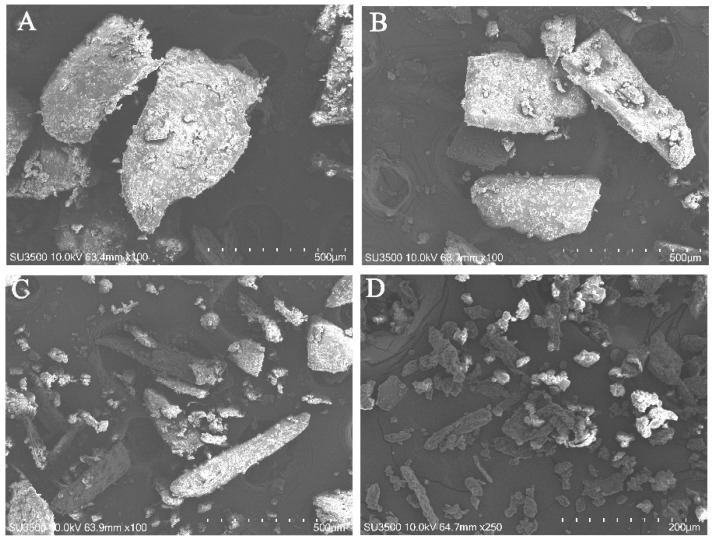
The Scanning electron microscopy (SEM) micrographs of the foxtail millet bran powder, with different particle sizes. (**A**) M60 100×; (**B**) M60–80 100×; (**C**) M80–120 100×; (**D**): M120 250×.

The SEM images of the millet bran powder with differently sized particles are presented in Figure 1. As the particle sizes of the millet bran decreased, their irregular shapes changed to spherical, while the surface roughness declined. The M60 sample (Figure 1A) was irregular and polygonal in shape, with a porous interior and a rough surface, while the M120 sample (Figure 1D) was smooth, with minimal porosity and rounded sides. The small particles aggregated, and the gap between the agglomerates increased (Figure 1D). The morphological changes in the millet bran powder were attributed to the strong mechanical forces during the grinding process, modifying the physicochemical properties accordingly [17].

**Figure 2:**
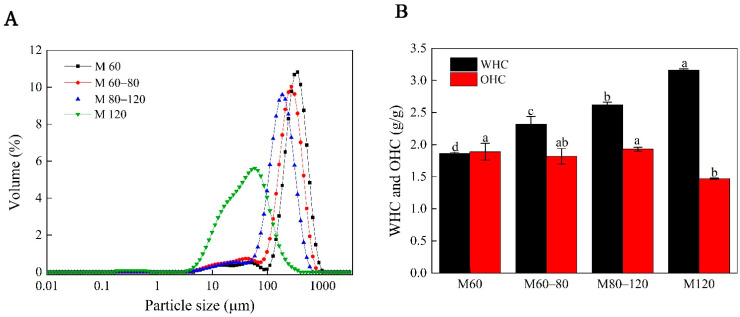
The particle size distribution: (**A**) water holding capacity (WHC) (**B**) and oil holding capacity (OHC) of the foxtail millet bran powder. The mean values denoted by different letters in the same column signify significant differences (*p* < 0.05).

The distribution curves denoting the particle sizes of the millet bran powder samples are illustrated in Figure 2, while the relevant parameters are listed in Table 1. The M60, M60–80, M80–120, and M120 curves gradually shifted to the left, indicating a particle size decline. Moreover, the M120 samples displayed a wider particle size distribution, which corresponded with the span value provided in Table 1. This observation corresponded with the findings of Li et al. [18] for chrysanthemum powder. In general, larger particles decreased the span value [19]. The average particle diameters (D_50_) of the four samples after pulverization were 330.3 μm (M60), 261.5 μm (M60–80), 187.0 μm (M80–120), and 54.7 μm (M120), respectively. Furthermore, the difference between the volume median (D_3,2_) and the weighted mean diameter (D_4,3_) of the surface area gradually decreased as the D50 declined. The Φ increased at stronger mechanical grinding levels, with M120 displaying the highest Φ value of 55.12%. The surface area would increase as the particle size became smaller. When D50 was below 100 μm, the particle size decrease led to a higher specific surface area, reflecting the increased potential of millet bran utilization for biomass hydrolysis, chemical extraction, and improved food bioavailability [9].

### 3.2. Physical Properties

The powder density can help facilitate food quality control [20]. The organic powder bulk density is dependent on the particle defects, closed pores, cracks, voids, and presence of a thin air film [21]. As illustrated in Table 1, the tap and bulk densities of the millet bran powder increased substantially, from 0.407 × 10^3^ kg/m^3^ to 0.512 × 10^3^ kg/m^3^ and from 0.294 × 10^3^ kg/m^3^ to 0.384 × 10^3^ kg/m^3^ as the particle sizes decreased. A finer powder indicated less pore space between the particles, increasing the density as the particle sizes declined [22]. Similar results were reported for the powder derived from Vaccinium bracteatum Thunb leaves [23]. However, He et al. [24] observed a different tendency for wheat bran powder; they found that smaller particles displayed lower bulk and tap densities. This may be attributed to the different ingredients in the organic powder, as well as the varied grinding methods. The θ value rose from 58.31° to 63.99°, in conjunction with a particle size decline, and α increased from 33.38° to 52.20°, indicating that the granular bulk exhibited lower flowability. This corresponded with the findings obtained by Zhang et al. [9] for tobacco leaf powder. However, contrary results were found in wheat bran [24]. This could be attributed to the fact that the high oil content in finer millet bran powder (Table 2, 14.20%) promoted particle agglomeration, forming a cone with a significant angle. In addition, the moisture levels in the powder substantially affected the α and θ [21]. No differences were evident between the moisture levels of the samples containing differently sized particles (Table 2).

The color analysis results are presented in Table 3. The M160 sample displayed the highest L* value, probably resulting from the considerable increase in the surface area, producing higher light reflection [25]. The a* and b* values decreased with a decline in the particle sizes of the powder, while redness and greenness changes were difficult to observe intuitively, due to small aberrations. Therefore, the a* value may not be the main factor affecting the color differences in the millet bran powder. After grinding, all samples displayed lower b* values. This might be due to the aggregation phenomenon of phenolic compounds and the degradation of chlorophyll [26]. The ΔE values increased significantly as the samples progressed from M80–120 to M 120 (*p* < 0.5). The results indicated that the color of the powder was brighter after grinding. Similar results were obtained for wheat bran and highland barley bran [24]. The reduction in particle size increased the surface area and exposed the internal structure of cellulose and hemicellulose, which affects the color of the powder. The color analysis revealed that fine millet bran powder might be utilized as ingredients.

As shown in Figure 2, the WHC increased from 1.86 g/g to 3.16 g/g as the sizes of the particles decreased from M60 to M120. The WHC indicated the capacity of the powder interstices to retain water. The hydration properties of the millet bran improved as the particles became smaller, due to a larger surface area, increased number of polar groups, and exposure of other millet bran powder water-binding sites to the surrounding water [27]. The M120 fraction had lower OHC, which could be ascribed to the fact that fine powders may increase the adsorption sites of the millet bran powder, consequently enhancing physical oil retention [17].

### 3.3. Crystalline Structure and Thermal Properties

**Figure 3:**
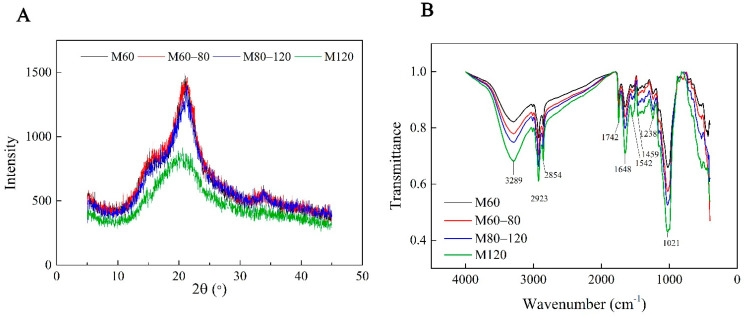
The X-ray diffraction (XRD) (**A**) patterns and Fourier-transform infrared (FTIR) (**B**) spectra of the foxtail millet bran powder with different particle sizes.

The XRD patterns and degree of crystallinity of the millet bran powder are illustrated in Figure 3. The millet showed a typical A-type polymorphic form [28] and a prominent 2θ peak at around 21.5°. No significant differences were evident in crystallinity as the particles declined from 292 μm (M60) to 186 μm (M80–120) (Table 4). However, when the particles decreased to 58.9 μm (M120), the crystallinity declined significantly, due to severe damage to the cellulose-chain hydrogen bonds in the cell walls [29].

Figure 3 presents the FTIR spectra of the millet bran powder with different particle sizes. These peaks were ascribed to the primary chemical constituents in the millet bran. The wide band at 3289 cm^−1^ was governed by the O-H stretching vibration in the fiber and polyphenol structures [30]. C-H group stretching was responsible for the 2923 cm^−1^ and 2854 cm^−1^ absorption bands, which typified polymers based on polysaccharides [31]. The 1742 cm^−1^ absorption was ascribed to the carbonyl group (C=O) stretching band [32], while the bands at approximately 1648 cm^−1^ and 1542 cm^−1^ denoted the respective stretching bands of aromatic C=O and N-H [32]. Due to the presence of tightly bound water, the absorption at 1459 cm^−1^ represented the C-H band [33]. The peaks at 1238 cm^−1^ could be attributed to C-O-C stretching vibration [34], while the absorption at 1021 cm^−1^ was characteristic of C-O bond stretching. This was consistent with the infrared spectra obtained for pearl millet and maize [33,35]. Overall, the FTIR patterns of the various millet bran powders were similar, while no additional chemical functional groups were evident, indicating that the techniques used for grinding did not substantially disrupt the primary constituent molecule structures. However, as the particles became smaller, the absorbance intensity rose significantly, indicating that the intramolecular cellulose, or hemicellulose, hydrogen bonds were broken to form new, amorphous cellulose and soluble saccharides by the mechanistic forces generated during the grinding process [8]. The results indicated that mechanical grinding exerted almost no effect on the primary molecular structure of the millet bran powder, instead destroying the polymer chain length [36]. A similar result was also reported for tobacco leaf and the stem [37].

The thermal properties of the millet bran powder samples were determined via DSC. The peak temperatures and gelatinization enthalpy are presented in Table 4. The endothermic peak (T_p_) at around 140–180 °C was attributed to bound water evaporation. The T_p_ values decreased as the particle sizes declined. This may be attributed to more moisture entering the interior of the powder after the particle size reduction, increasing the evaporation energy. This indicates that the powder with small particles can be utilized at higher temperatures [24]. In addition, the endothermic enthalpy changes (ΔH) declined from 31.21 J/g to 12.90 J/g as the particle sizes became smaller, indicating that less energy was expended to break the non-covalent bonds. This is possibly due to high temperatures generated by the vigorous, extended grinding process [20].

### 3.4. Functional Properties

Table 2 shows the impact of the particle sizes on the nutritional characteristics and antioxidant ability. The results revealed that smaller particles significantly changed the constituents of the millet bran powder. The protein, fat, and carbohydrate content increased as the millet bran-powder particles decreased. A comparable increase in the protein content with a decline in the sizes of the particles was reported for hard wheats by Siliveru et al. [38], which was ascribed to the characteristics of the cell content and cell walls. This led to breaks at the most vulnerable points along the cell walls or via starch granules, generating smaller starch granule fractions entrenched in the protein matrix. The decline in the particle size facilitated a considerable increase in the fat content in the millet bran powder. The fine powder increased the surface area available to the solvent during fat extraction [39]. The TDF decreased from 72.61% to 41.76%, with a decline in the particle size, which was due to lignin, cellulose, and hemicellulose degradation into smaller molecular components [20]. Moreover, the IDF content decreased and the SDF increased, indicating a transition from the insoluble to the soluble part [40]. The total phenolic content in the millet bran powder tended to increase with decreased particle size [41], which is attributed to the destruction of the millet bran cell wall structure, such as cellulose, during the milling process, and the small particle size resulted in a large contact area and a short transfer path during the extraction of total phenolics. Similar results were obtained for rice bran, wheat bran [17], and Qingke bran [40]. The surfaces of the bran particles increased as the particles became smaller, while the phytochemicals, including phenolics, embedded in the fibrous matrix were rapidly released [17]. The antioxidant capacity showed the same trend as the change in the total phenolic content (Table 2). Furthermore, the M120 sample exhibited higher DPPH and ABTS antioxidative ability, which was ascribed, in part, to more significant total phenolic extraction [9]. These results are in accordance with the research of Mustac et al. [42], who reported that micronization could enhance the antioxidant activity of proso millet bran.

## 4. Conclusions

This study investigates the effect of the sizes of particles on the functional, physicochemical, and structural characteristics of millet bran powder. The fine powder (M120 samples) exhibits a higher bulk density, WHC, tap density, θ, α, and peak temperature (T_p_), and a lower OHC and CI value. Moreover, the fine millet bran powder presents higher protein, fat, SDF, total phenolic content, and antioxidant properties than its coarse counterpart. The foxtail millet bran can be utilized as a potential resource of phenolic antioxidants. The current study helps to determine the appropriate grinding particle size range for mechanical grinding pretreatment of millet bran for utilization. Further research should address the functional activity and nutrient bioavailability of millet bran-enriched foods to better utilize cereal by-products contributing to economics and the sustainability of the cereal chain.

## Figures and Tables

**Table 1:** The particle size distribution of the foxtail millet bran powder.

Sample	D_10_ (μm)	D_50_ (μm)	D_90_ (μm)	D_3,2_ (μm)	D_4,3_ (μm)	Specific Surface Area (m^2^/kg)	Span	Cell Wall Breakage Ratio (%)
M60	141.3 ± 7.9 ^a^	330.3 ± 3.8 ^a^	579.7 ± 3.0 ^a^	145 ± 4.0 ^a^	346.3 ± 3.8 ^a^	41.50 ± 1.15 ^b^	1.33 ± 0.03 ^c^	8.81 ± 0.17 ^d^
M60–80	73.8 ± 0.8 ^b^	261.5 ± 0.5 ^b^	471.5 ± 1.5 ^b^	115.5 ± 0.5 ^b^	273.5 ± 0.5 ^b^	50.62 ± 1.14 ^b^	1.52 ± 0.01 ^b^	11.04 ± 0.03 ^c^
M80–120	79.2 ± 0.7 ^b^	187.0 ± 1.0 ^c^	349.5 ± 0.5 ^c^	108.5 ± 1.5 ^b^	202.5 ± 0.5 ^c^	52.67 ± 0.59 ^b^	1.45 ± 0.01 ^b^	15.20 ± 0.11 ^b^
M120	11.8 ± 0.1 ^c^	54.7 ± 0.4 ^d^	116.3 ± 2.3 ^d^	17.0 ± 0.3 ^c^	55.6 ± 0.9 ^d^	335.83 ± 6.17 ^a^	2.45 ± 0.03 ^a^	55.12 ± 0.61 ^a^

D_10_: 10% of the volume that is smaller than the size indicated; D_50_: 50% of the volume that is smaller than the size indicated; D_90_: 90% of the volume that is smaller than the size indicated; D_3,2_: the volume median; D_4,3_: the weighted mean diameter; the mean values denoted by different superscripts in the same row signify significant differences (*p* < 0.05).

**Table 2:** The nutritional characteristics and antioxidant activity of the foxtail millet bran powder.

Components	M60	M60–80	M80–120	M120
Moisture content (%)	5.13 ± 0.03 ^a^	5.14 ± 0.04 ^a^	5.12 ± 0.04 ^a^	5.14 ± 0.01 ^a^
Protein (%)	5.57 ± 0.10 ^d^	6.20 ± 0.05 ^c^	7.71 ± 0.25 ^b^	17.20 ± 0.07 ^a^
Fat (%)	5.40 ± 0.07 ^d^	5.80 ± 0.00 ^c^	7.40 ± 0.14 ^b^	14.20 ± 0.14 ^a^
Carbohydrates (%)	3.63 ± 0.73 ^c^	5.12 ± 0.76 ^c^	9.88 ± 0.14 ^b^	14.19 ± 0.63 ^a^
TDF (%)	72.61 ± 0.47 ^a^	70.19 ± 0.70 ^b^	62.49 ± 0.02 ^c^	41.76 ± 0.54 ^d^
IDF (%)	72.00 ± 0.64 ^a^	69.50 ± 0.57 ^b^	62.00 ± 0.14 ^c^	40.60 ± 0.57 ^d^
SDF (%)	0.49 ± 0.12 ^b^	0.66 ± 0.17 ^b^	0.69 ± 0.13 ^b^	1.16 ± 0.02 ^a^
Total phenolic (μg Trolox/g)	5.57 ± 0.10 ^d^	6.20 ± 0.05 ^c^	7.71 ± 0.25 ^b^	17.20 ± 0.07 ^a^
DPPH (μg Trolox/g DW)	5.40 ± 0.07 ^d^	5.80 ± 0.00 ^c^	7.40 ± 0.14 ^b^	14.20 ± 0.14 ^a^
ABTS (μg Trolox/g)	3.63 ± 0.73 ^c^	5.12 ± 0.76 ^c^	9.21 ± 0.04 ^b^	15.34 ± 0.65 ^a^

TDF: total dietary fiber; IDF: insoluble dietary fiber; SDF: soluble dietary fiber; DPPH: DPPH radical scavenging activity; ABTS: ABTS scavenging activity; the mean values denoted by different superscripts in the same column signify significant differences (*p* < 0.05).

**Table 3:** The bulk and tap density, θ, α, and color of the different foxtail millet bran powders.

Properties	M60	M60–80	M80–120	M120
Bulk density (10^3^ kg/m^3^)	0.294 ± 0.003 ^d^	0.312 ± 0.004 ^c^	0.341 ± 0.004 ^b^	0.384 ± 0.007 ^a^
Tap density (10^3^ kg/m^3^)	0.407 ± 0.008 ^d^	0.435 ± 0.007 ^c^	0.486 ± 0.004 ^b^	0.512 ± 0.007 ^a^
Angle of repose (°)	58.31 ± 0.32 ^b^	59.82 ± 0.29 ^b^	62.61 ± 0.37 ^a^	63.99 ± 0.56 ^a^
Angle of slide (°)	33.38 ± 1.15 ^c^	41.30 ± 0.51 ^b^	49.47 ± 0.59 ^a^	52.20 ± 0.94 ^a^
L*	61.71 ± 0.16 ^c^	63.39 ± 0.15 ^b^	63.52 ± 0.19 ^b^	69.70 ± 0.05 ^a^
a*	7.23 ± 0.01 ^a^	6.75 ± 0.04 ^b^	6.47 ± 0.09 ^c^	4.57 ± 0.03 ^d^
b*	32.32 ± 0.01 ^a^	31.23 ± 0.20 ^b^	30.68 ± 0.18 ^c^	26.01 ± 0.10 ^d^
ΔE	0	2.10 ± 0.15 ^b^	2.56 ± 0.28 ^b^	10.51 ± 0.10 ^a^

L*: brightness of samples; a*: redness of samples; b*: yellowness of samples; ΔE: total color difference. The mean values denoted by different superscripts in the same row signify significant differences (*p* < 0.05).

**Table 4:** The crystallinity, peak temperature, and enthalpy of the foxtail millet bran powder.

Sample	Crystallinity (%)	T_p_ (°C)	ΔH (J/g)
M60	42.2 ± 2.9 ^b^	145.0 ± 9.3 ^b^	31.2 ± 0.1 ^a^
M60–80	41.3 ± 2.4 ^b^	148.9 ± 17.1 ^ab^	21.2 ± 4.1 ^ab^
M80–120	38.9 ± 1.7 ^b^	175.3 ± 5.2 ^a^	18.3 ± 5.8 ^b^
M120	22.0 ± 2.3 ^a^	176.7 ± 2.3 ^a^	12.9 ± 4.9 ^b^

T_p_: peak temperature; ΔH: enthalpy. The mean values denoted by different superscripts in the same row signify significant differences (*p* < 0.05).

## Data Availability

The data presented in this study are available in the article.
